# Complete Genome Sequence of the Model Halovirus PhiH1 (ΦH1)

**DOI:** 10.3390/genes9100493

**Published:** 2018-10-12

**Authors:** Mike Dyall-Smith, Felicitas Pfeifer, Angela Witte, Dieter Oesterhelt, Friedhelm Pfeiffer

**Affiliations:** 1 Computational Biology Group, Max-Planck-Institute of Biochemistry, Am Klopferspitz 18, 82152 Martinsried, Germany; mike.dyallsmith@gmail.com (M.D.-S.); oesterhe@biochem.mpg.de (D.O.); 2 Veterinary Biosciences, Faculty of Veterinary and Agricultural Sciences, University of Melbourne, Parkville, VIC 3052, Australia; 3 Department of Biology; Microbiology and Archaea, TU Darmstadt, Schnittspahnstrasse 10, 64287 Darmstadt, Germany; pfeifer@bio.tu-darmstadt.de; 4 Department of Microbiology, Immunobiology and Genetics, MFPL Laboratories, University of Vienna, Dr. Bohr-Gasse 9, Vienna 1030, Austria; angela.witte@univie.ac.at

**Keywords:** halovirus, virus, halophage, *Halobacterium salinarum*, Archaea, haloarchaea, halobacteria, genome inversion

## Abstract

The halophilic myohalovirus *Halobacterium virus phiH* (ΦH) was first described in 1982 and was isolated from a spontaneously lysed culture of *Halobacterium salinarum* strain R1. Until 1994, it was used extensively as a model to study the molecular genetics of haloarchaea, but only parts of the viral genome were sequenced during this period. Using Sanger sequencing combined with high-coverage Illumina sequencing, the full genome sequence of the major variant (phiH1) of this halovirus has been determined. The dsDNA genome is 58,072 bp in length and carries 97 protein-coding genes. We have integrated this information with the previously described transcription mapping data. PhiH could be classified into Myoviridae Type1, Cluster 4 based on capsid assembly and structural proteins (VIRFAM). The closest relative was *Natrialba* virus phiCh1 (φCh1), which shared 63% nucleotide identity and displayed a high level of gene synteny. This close relationship was supported by phylogenetic tree reconstructions. The complete sequence of this historically important virus will allow its inclusion in studies of comparative genomics and virus diversity.

## 1. Introduction

The temperate myovirus *Halobacterium virus phiH* (ΦH) infects the extremely halophilic archaeon *Halobacterium salinarum* strain R1 (DSM 671) and was isolated after the spontaneous lysis of a culture of its host [1]. Purified virions require 3.5 M NaCl for stability, have an isometric head of 64 nm diameter and a long, contractile tail (170 × 18 nm) with short tail fibres [1,2]. Virus preparations contain 3 major and 10 minor proteins [3]. The virus genome is linear dsDNA with a G+C content of 64%, contains a *pac* site, is about 3% terminally redundant and partially circularly permuted, and estimated to be 59 kb in length [4,5]. In the provirus state, the genome is extrachromosomal, covalently closed and circular, and 57 kb in length [4]. While always classified within the *Myoviridae*, the genus name has changed over the years from phiH-like viruses to *Phihlikevirus*, and most recently to *Myohalovirus* [6,7]. The species name itself has changed from *Halobacterium phage phiH* to *Halobacterium virus phiH* [6,7] but for convenience we will refer to it from here onwards simply as phiH, and the analysed variant as phiH1 or halovirus phiH1.

The original lysate of phiH was found to consist of a mixture of several distinct variants that appeared to have arisen from the activity of insertion sequences. The predominant variant, phiH1, was plaque-purified and a restriction map determined [5]. This was used for further study [3]. PhiH1 became a key model in the study of gene expression and regulation in haloarchaea and was instrumental in the development of genetic tools and methods in these extremophiles. Examples include the polyethylene glycol (PEG)-mediated transfection method [8], the pUBP1 cloning/expression vector [9], the identification of archaeal promoters, mapping transcription start and stop sites [10] and the analysis of gene regulation via repression [11,12]. The presence and function of antisense RNA in haloarchaea was first described in this virus [13]. An 11 kb invertible segment of the virus genome, called the L-region, was found to be flanked on one or both sides by the insertion sequence ISH1.8, and could also circularize to form a 12 kb plasmid (including one copy of ISH1.8), with subsequent loss of the remaining phage DNA [14]. A strain carrying this plasmid was immune to infection [14]. 

Unfortunately, work on phiH stopped in 1994 [15,16] but a related virus, *Natrialba* virus phiCh1 (φCh1), was described a few years later [17] and continues to be studied in the Witte laboratory [18,19]. PhiCh1 infects a haloalkaliphilic archaeon, *Natrialba magadii*, and the genomes of both host and virus are fully sequenced [18,20]. The provirus state of phiCh1 corresponds to plasmid pNMAG03 carried by *Nab. magadii*. A full comparison between phiCh1 and phiH1 was prevented as only parts of the phiH genome were ever determined. This deficit also prevented the inclusion of phiH in broad-ranging studies of virus diversity, taxonomy, and evolution. The aim of this study was to complete the phiH1 genome sequence and provide a thorough annotation. This will not only provide a better understanding of the results from previous studies on this virus but also allow complete genomic comparisons with a wealth of other datasets, including other sequenced viruses, haloarchaeal proviruses, metaviromic/metagenomic and environmental RNA sequences.

## 2. Materials and Methods

### 2.1. Virus DNA and Sequencing Methods

Purified phiH1 DNA [1] was originally provided to F. Pfeifer by Hans-Peter Klenk while both were working in the department of W. Zillig [21]. The DNA was stored frozen at −80 °C until use. Sequencing was performed in two stages. For the first stage, all available sequences of phiH1 were downloaded from National Center for Biotechnology Information (NCBI) [22] and imported into the Phred–Phrap–Consed package [23]. Overlapping sequences were assembled and primers designed to gather additional sequences using Sanger technology. This consisted either of primer-walking directly on virus DNA, or on polymerase chain reaction (PCR) amplimers, or PCR-sequencing across gaps. The resulting sequence reads were progressively assembled into contigs, base calls inspected manually and corrected where needed, and new primers designed for further rounds of sequencing until all gaps were closed. Except for overlaps, this approach left most of the previously published sequences unchecked.

In the second stage, short-read Illumina HiSeq sequencing of phiH1 DNA was performed (Max-Planck Genome Centre, Cologne, Germany). This returned 243 Mb of high quality sequence data (coverage = 4200-fold). De-novo assembly did not produce a single contig, due to short read-lengths and the presence of repeat sequences within the viral genome, but reads could be confidently mapped to the genome sequence obtained in the first stage (*Map to Reference* option; Geneious mapper method) in order to improve the sequence reliability.

### 2.2. CRISPR Spacer Searches

The crass v0.3.12 software [24] was used to extract CRISPR spacer sequences from genomic/metagenomic data available at the NCBI SRA database (accessed 27 July 2018) [25], as described previously [26]. These included all available genomes of members of the class Halobacteria, and metagenomes of hypersaline environments. CRISPR direct repeats (DR) identified by crass were used to search the CRISPRfinder database (accessed 25 July 2018) [27] for haloarchaea with matching or closely matching DR. 

### 2.3. Bioinformatic Methods 

Gene annotation used a combination of gene prediction with GeneMarkS-2 [28] and manual refinement using database searches (BLASTp/BLASTn; nr databases) at the NCBI webserver [29]. Repeats were identified by BLASTn, dot-plot comparison in Yass [30], and with tools within the Geneious software suite [31]. Circos plots were performed via the circoletto webserver [32]. Plots are coloured by the ‘score/max’ ratio of tBLASTx bitscores (real score/maximal score). Colours are: blue ≤ 0.25, green ≤ 0.50, orange ≤ 0.75, red > 0.75. Sequence mapping, alignments, editing and phylogenetic tree reconstructions were performed with Geneious software version 10.2 [31]. For phylogenetic tree reconstructions, protein sequences were first aligned using CLUSTALW, and trees inferred using the Neighbor-Joining algorithm (within Geneious). Consensus trees were determined after 100 bootstrap repetitions. Protein structural modelling used the I-Tasser webserver [33]. Identification of the *pac* site utilised the program PhageTerm [34] as implemented on the CPT Phage Galaxy [35]. The VIRFAM webserver [36] uses proteins of the phage head-neck-tail module to cluster phages into related groups, and was used to classify phiH1. 

### 2.4. Data Availability

The phiH1 genome sequence has been deposited at Genbank under the accession MK002701. Raw reads were submitted to the SRA archive under accession SRP159490.

## 3. Results and Discussion

### 3.1. Sequence and Annotation of PhiH1

**Figure 1:**
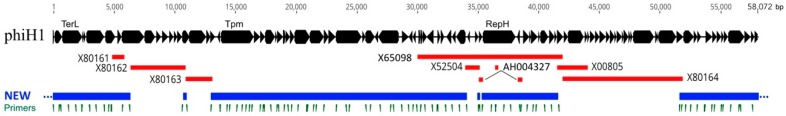
Diagram of the phiH1 genome with lines below showing regions previously sequenced (red) along with their database accessions. The blue lines (NEW) indicate regions sequenced in the present study by Sanger sequencing. Tick marks (dark green) below the blue lines show the positions of oligonucleotide primers used for PCR and primer-walking. Dots at the right and left contig ends indicate sequence continuity between them. Scale bar at top shows position in bp.

The previously sequenced regions of the phiH1 genome represented about 50% of the complete sequence (Figure 1, red lines). Using virus DNA as template, the gaps between these sequences were PCR amplified and Sanger sequenced. However, the quality of the previously sequenced regions was of uncertain reliability. High-coverage Illumina sequencing (ca. 4200-fold) was then used to enhance sequence confidence. Sequence revisions were only found to be required in previously deposited sequences but not to Sanger sequencing results of the first stage of the project. While the virus DNA found in capsid particles is linear, the head-full packaging process produces a population of molecules that are terminally redundant and partially circularly permuted [1]. The complete genome sequence determined in the current study is represented as the provirus form; a circular sequence of 58,072 bp. This value is close to the published size of 57 kb, estimated from restriction fragment sizes [4,37]. The G+C content of the genome was 63.7%, almost identical to the published value of 64% [3] but slightly lower than that of the host chromosome (68.0%) [38].

The original restriction map of phiH1 DNA, as determined by [5], corresponded closely with the *in silico* map inferred from the phiH1 genome sequence (Figure S1). The *pac* site located at the left end of the restriction map matched closely to the corresponding *pac* sequence of phiCh1. While the *pac* site of phiCh1 had been localized by restriction mapping [18], it had not been precisely mapped. For consistency, the start point of phiH1 was set to the corresponding start of phiCh1 even though this splits the *terS* gene. Using this numbering, the program PhageTerm [34] was used to analyse the mapping of Illumina reads to the phiH1 genome, and this located the *pac* site terminal base at nt 46, with high probability (*p* = 2.5 × 10^−238^). This is within the *terS* coding sequence (CDS) close to the stop codon and within a GC-rich region that is strongly conserved between phiH1 and phiCh1. 

**Figure 2:**
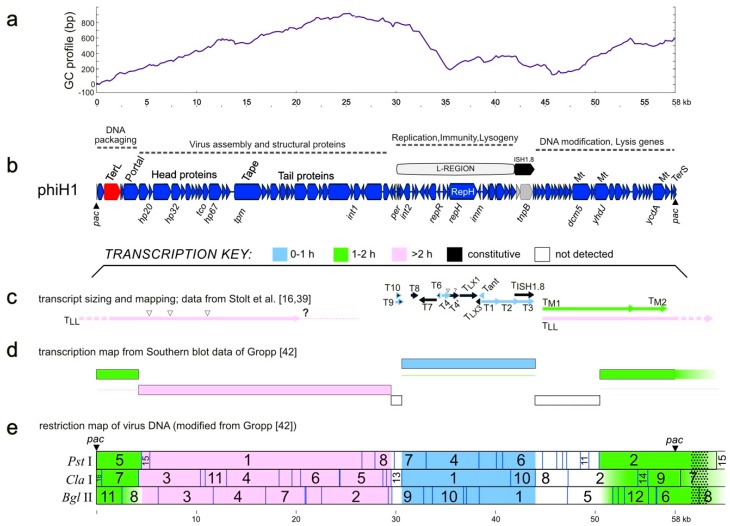
PhiH1 GC-profile, genetic map, and corresponding transcription program (adapted from [16,42]). (**a**) GC-profile of the phiH1 genome. (**b**) Genetic map of the phiH1 genome, showing coding sequences as red, blue or grey arrows. Dotted lines above indicate gene clusters involved in particular functions. Some CDS are labelled above the map, e.g., TerL, terminase large subunit; Portal, portal protein; Tape, tape-measure protein; RepH, replicase (label within CDS arrow); Mt, DNA methylases; TerS, terminase small subunit. Some genes are shown below the map, such as *hp32*, encoding the major capsid protein HP32. Panels c, d and e summarise transcription data from previously published studies, and above them is a colour key that indicates the time of appearance of early (0–1 h, blue), middle (1–2 h, green) and late (>2 h, pink) transcripts. (**c**) Precise mapping of viral transcripts, including start and termination sites [16,39]. (**d**) Summary transcription program of lytic infection based on hybridisation of labelled infected-cell transcripts to restriction fragments of virus DNA [42]. Thin coloured lines indicate whether continuing transcription persists over time. (**e**) The transcription map data of [42] are shown, projected onto the in silico restriction map of phiH1, as determined from the complete genome sequence (this study). Enzymes are indicated at the left. Numbers on the restriction map refer to those of the original publication of [42] (see also Figure S1). Coloured shading follows that of panels c and d. Dotted pattern shown beyond the right-hand *pac* site indicates terminal redundancy of virus DNA. Scale bars (in kb) are shown below panels a and e.

Annotation of the phiH1 genome resulted in 97 CDS (Table 1), most of which were encoded on the plus strand (86/97, Figure 2 panel b), and were frequently closely spaced, with 45 overlapping at start/stop codons and 23 separated by ≤8 nt. Many genes were in functional groupings typical of bacteriophages (Figure 2 panel b). The left end of the genome encodes DNA packaging proteins (e.g., terminase, portal protein), then virus assembly and structural proteins (e.g., major capsid protein, tape-measure protein, tail proteins). The three main proteins of purified virus were originally labelled by their estimated sizes on sodium dodecylsufate (SDS)-polyacrylamide gels (22, 53 and 80 kDa) [1], which were later revised to 27, 46 and 80 kDa [3] but in 1994, Stolt et al. [39] determined their N-terminal amino acid sequences and used this information to map the proteins (HP20, HP32 and HP67) to their genes (*hp20*, *hp32*, *hp67*) and sequence them. The inferred molecular weights (MWs) of proteins HP20 and HP67 were noted by these authors to be much smaller than previous estimates. In the present study, the locations of these genes on the full genome sequence have been resolved, an error in the *hp20* (accession X80161) coding sequence was corrected, and the MWs of the inferred proteins calculated (11.6, 35.4 and 45.5 kDa). For consistency we have retained the original gene names (Table 1).

The next genomic region is a replication/regulatory module (the L-region) that encodes RepR (repressor), a ParA-family protein (partition) and RepH (replication). There is also a VapC-like protein that together with the small overlapping upstream CDS may form a toxin‒antitoxin pair that could be involved in plasmid maintenance [40]. The right end of the genome carries many genes with unknown function but includes genes specifying DNA methylases and cell lysis proteins. The taxonomic position of phiH1 was assessed using the VIRFAM webserver [36], which classifies bacteriophages and archaeal viruses based on the order and similarity of capsid assembly/structural proteins. Consistent with previous studies [6], phiH1 was classified by this system as a member of the Myoviridae (Type1, Cluster 4).

A GC-profile plot [41] of the phiH1 genome shows a major low point inflection within the L-region (Figure 2, panel a), indicating a potential replication origin. The L-region is ~12 kb in length, can replicate as a plasmid in *Halobacterium* [14], and carries genes encoding a replication protein (RepH), and a DNA-binding repressor (RepR). It can also provide cells with immunity to infection by phiH1 virus. The transcription program of phiH1 during lytic growth (panels c, d and e) has been summarized from previous studies, and shows temporal changes (early, middle and late transcripts). The broad directions of transcription reflect the closely spaced and similarly directed gene clusters as well as the correspondence with functional gene groupings (panel b). The lowest two panels (d, e) summarize the results of hybridizing labelled transcripts from infected cells to Southern blots of restricted phiH1 DNA [42], so mapping transcripts to fragments of the virus genome. Panel c shows a summary of the virus-specific transcripts that were sized by agarose gel electrophoresis and had 5′ start sites mapped. While transcription across the L-region has been examined in more detail compared to the rest of the genome, there remains much that is incomplete or uncertain. For example, the 3′ end of the late transcript labelled T_LL_, which is depicted ending in a dotted line and question mark (at ~21 kb), has not been determined. This transcript could potentially extend for another 5.5 kb. Counter-transcripts are commonly produced by prokaryotes and their viruses and play important roles in gene regulation. Their presence and activity in phiH1 gene expression has been studied and was one of the first reports of antisense RNA in Archaea [13]. However, this interesting topic remains to be fully explored.

Corrections to the previously sequenced regions resulted in significant changes to several coding sequences. For example, the *tnpB* gene of transposon ISH1.8 (nt 41,906–43,789) was thought to be inactive as it was split into three CDS by multiple mutations [43]. The high-quality Illumina sequence data show, however, that the gene is intact and that the previously reported transposon ISH1.8 (X00805) is actually an exact copy of transposon ISH12 from the host *Hbt. salinarum* strain R1 [44]. The element plays a key role in the mobilisation of the L-region of the genome to form the 12 kb plasmid, pΦHL [14,43]. Another case is the Dcm5 cytosine methylase, which was also reported as being split [39]. The revised sequence shows that the gene codes for a single, probably functional protein (PhiH1_405, nt 47,732–49,618) and not for the two parts (dcm5a, dcm5b) as previously reported. Although phiH1 carries three potentially active DNA methylase genes (*dcm5*, *yhdJ* and *ycdA*), the presence of modified bases in phiH1 DNA was not detected in the chromatographic (high-pressure liquid chromatography) profiles of deoxyribonucleosides released by enzymatic hydrolysis [45]. In that study, the genomes of phiH and another, unrelated virus (phiN) were analysed, and while unmodified dC was detected in phiH, the phiN genome contained only methylated dC (Figure 3 in [45]). The related halovirus phiCh1 carries homologs of two of the phiH1 methylases, and one of them, N6-adenine methylase (ORF94/M·φCh1-I, corresponding to YcdA of phiH1) has been shown to methylate DNA at GATC motifs [46] but the proportion of sites found to be modified in virus DNA by M·φCh1-I varies from 5% to 50%, depending upon the infection conditions. Modifying only some of the available sites is presumably advantageous to avoid host restriction, as distinct enzymes may target either unmethylated or methylated sites. 

### 3.2. Matches to CRISPR Spacers 

The phiH1 genome was used to search for matching CRISPR spacers among metagenomic datasets of hypersaline environments downloaded from the NCBI sequence read archive (SRA; see methods). Only four spacers showing close to moderate similarity to phiH1 were detected (Table 2). These spacers match to virus genes encoding structural and non-structural proteins, and the DRs of these spacers show that they are carried by haloarchaea. The datasets include metagenomes from the USA and Iran, as well as an isolate from the Andaman Islands, India. The results suggest that phiH1-like viruses are geographically widespread.

### 3.3. Relatives and Phylogeny of PhiH1

**Figure 3:**
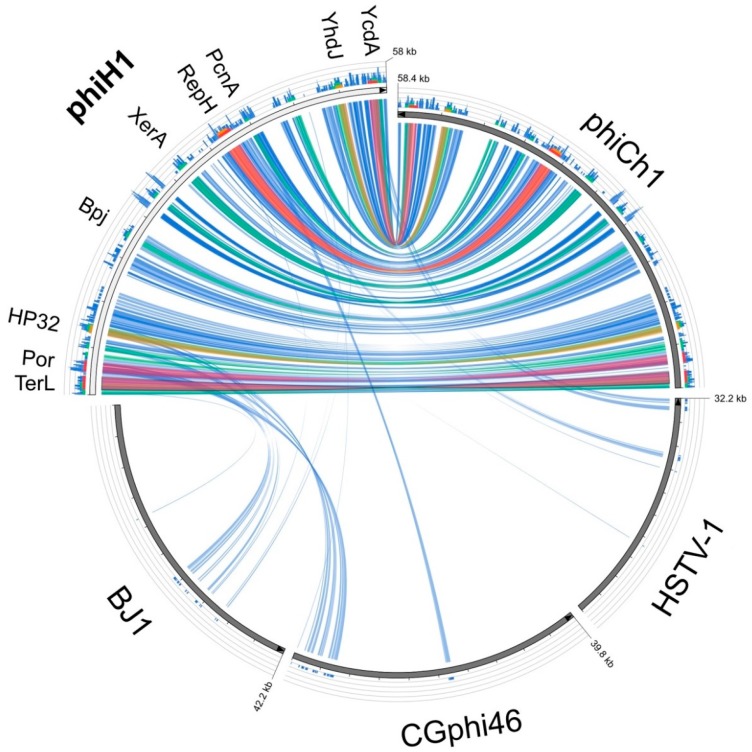
Circos plot of amino acid similarity (tBLASTx) between phiH1 and the haloviruses phiCh1, BJ1, CGphi46 and HSTV-1. The threshold for connecting lines was E-value ≤ 10^−40^, with line colours reflecting the ratio of actual tBLASTx bitscore to the maximal score (using ‘score/max’ ratio colouring with blue ≤ 0.25, green ≤ 0.50, orange ≤ 0.75, red > 0.75). The outer histogram counts how many times each colour has hit the specific part of the sequence and uses an equivalent colouring scheme. The distance between successive tick marks shown along each virus genome represents 0.1 of the full genome length. Protein names shown along the phiH1 genome indicate the positions of the corresponding genes.

The only close matches to the phiH1 genome in the GenBank database were phiCh1 and the corresponding *Nab. magadii* plasmid pNMAG03 (BLASTn, accessed 20 July 2018). A dot-plot comparison of phiH with phiCh1 (Figure S2) revealed a largely colinear relationship (green line) and an overall nucleotide similarity of 63%. The plot also highlights several indels (line gaps) and two regions showing inversions (red lines). Inversion 1 (nt 24,227–27,767) corresponds closely in sequence and arrangement to the invertible region described in phiCh1 (ORF34-36) that has a central XerD type integrase/recombinase gene flanked by inverted repeats, and facilitates switching between two related tail fibre genes, each containing numerous short repeats [18]. The phiH1 orthologous integrase is PhiH1_175. In the current sequence version, PhiH1_165 is active while PhiH1_185 is uncoupled from a start codon and thus is inactivated. Upon inversion of the genome segment, PhiH1_185 is activated while PhiH1_165 is inactivated. Overall, this results in tail fibre protein switching, which may affect receptor binding specificity and host range of phiH1. The similarity in the tail fibre protein repeats is high enough to be detectable at the DNA level, which results in the X-shaped pattern for this region in the dot-plot. Inversion 2 (nt 31,932–34,126) occurs within the phiH1 L-segment, encompasses four CDS including a ParA-domain protein, and is nearby a different integrase/recombinase gene (Int2, PhiH1_240). Protein searches (BLASTp) of the phiH1 genome returned matches to phiCh1, a limited number of haloarchaeal genomes (often 5–10, which may flag proviral regions) and the haloarchaeal caudoviruses BJ1 [47] and CGphi46 (NC_021537), both of which infect *Halorubrum* spp. Pairwise alignments (BLASTp) between all phiH1 and matching phiCh1 proteins gave an average protein sequence identity of 70% (range 39–95%; with a few exceptions, see footnote 5, Table 1). Figure 3 is a graphical comparison of phiH1 proteins (tBLASTx) with those of phiCh1, BJ1, CGphi46 and, as an outlier, HSTV-1. The *Haloarcula* caudovirus HSTV-1 [48] shows very low similarity to phiH1. The figure summarizes the close similarity of phiH1 and phiCh1 proteins. BJ1 and CGphi46 show far fewer matching regions, mainly to proteins encoded near the left end of the phiH1 genome, a region specifying portal and capsid proteins. The three significant matches to HSTV-1 were to a methyltransferase (HSTV1_52), a hypothetical protein (HSTV1_53), and a DNA polymerase sliding clamp protein (HSTV1_40).

**Figure 4:**
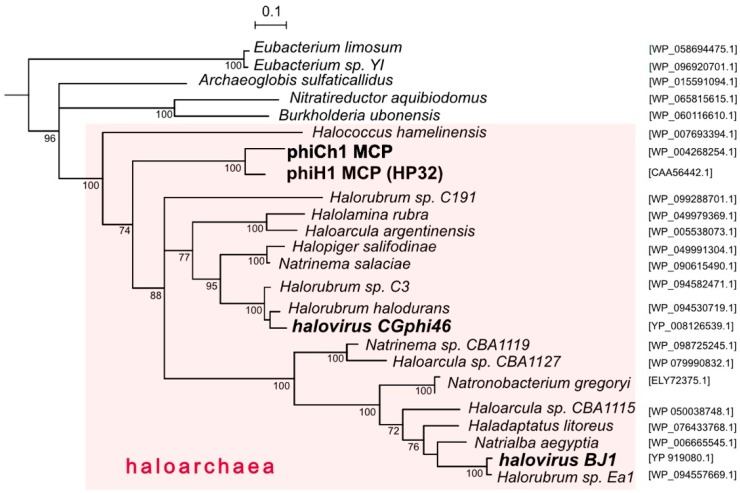
Phylogenetic tree reconstruction (NJ method) of major capsid proteins (MCP) of phiH1, other haloviruses and related proteins of haloarchaea. Species names of haloarchaeal species are shown, with accession numbers given at the right side. Bootstrap confidence values (100 repetitions) are shown at branch points. The pink shading highlights taxa belonging to the class Halobacteria. Scale bar (expected changes per site) is shown at top. The outgroup (not shown) consisted of distantly related MCP sequences of *Bacillus* spp. (WP_001060157.1, WP_098773561.1, WP_001064748.1 and WP_000178926.1).

While several caudovirus proteins have been used to infer virus phylogenies, the major capsid protein (MCP) is often used because of its functional constraints maintaining a conserved structure [49]. Figure 4 shows a tree reconstruction using an alignment of phiH1 MCP (HP32, 35.4 kDa) and related sequences. Haloarchaeal proteins are seen to branch together (pink shading) and within this cluster the phiH1 and phiCh1 MCPs form a distinct and closely branching clade. These two proteins share 82% amino acid identity. The MCPs of CGphi46 and BJ1 branch at distant locations from each other and from phiH1 MCP. Structures of close homologs of phiH1 HP32 have not yet been determined. However, the major capsid proteins of bacterial caudoviruses and eukaryotic herpesviruses share a common folding structure, the archetype of which is the phage HK97 MCP (gp5) [50]. Consistent with this, modelling of the phiH1 MCP (I-Tasser) returned bacteriophage HK97 gp5 (PDB 2fs3A) as the closest matching structure (Template Modeling (TM)-score = 0.848, Root-Mean-Square Deviation (RMSD) = 1.17). Based on structure prediction and homology modelling, the HK97-fold may also be present in the MCP of phiCh1 [49]. The structure of the MCP of the haloarchaeal podovirus, HSTV-1, has recently been shown to be of the HK97 type [48]. 

PhiH1 and phiCh1 display a close sequence similarity across most of their genomes yet infect physiologically and biochemically different haloarchaeal hosts. *Hbt. salinarum* is a widely distributed neutrophilic heterotroph with glycolipid-containing membranes, and has often been isolated from spoilage of salted products while *Nab. magadii* is a haloalkaliphile (optimum pH 9.5) that lacks glycolipids [51] and is restricted in its distribution to highly alkaline salt lakes [52]. Looking more widely, the presence of phiH1 MCP homologs in diverse genera of haloarchaea and two haloviruses (Figure 4) indicates that the *Myohalovirus* genus and related viruses are a highly successful group, the reasons for which are worthy of more detailed study, particularly when large-scale cultivation of *Halobacterium* becomes more common [53]. PhiH1 has been well studied in the past, and the completion of its genome sequence now allows it to be included in much of the sequence-based studies used today, including comparative virology, detection of proviruses in archaeal genomes, virus evolution and the microbial ecology of hypersaline environments.

## Figures and Tables

**Table 1:** Annotated coding sequences (CDS) of halovirus phiH1.

Start (nt)	Stop (nt)	Locus_tag	Length (bp)	Direction	Gene	Product	*Homologs*^1^: phiCh1, ORF pNMAG03 [Other]
115	717	PhiH1_005	603	+	-	uncharacterized protein	PhiCh1p02, ORF1 Nmag_4251
710	2371	PhiH1_010	1662	+	*terL*	terminase large subunit TerL	PhiCh1p03, ORF2 Nmag_4252
2377	2505	PhiH1_015	129	+	-	uncharacterized protein	Nmag_4253
2498	2689	PhiH1_020	192	+	-	uncharacterized protein	PhiCh1p05, ORF4 Nmag_4255
2686	4242	PhiH1_025	1557	+	*por*	portal protein Por	PhiCh1p07, ORF6 Nmag_4257
4246	5187	PhiH1_030	942	+	-	head morphogenesis protein	PhiCh1p08, ORF7 Nmag_4258
5261	5587	PhiH1_035	327	+	*hp20*	capsid protein HP20	[AJF28118.1]
5667	7466	PhiH1_040	1800	+	-	prohead protease	^4^ PhiCh1p09, ORF8 ^4^ PhiCh1p10, ORF9 Nmag_4259
7506	8468	PhiH1_045	963	+	*hp32*	major capsid protein HP32	PhiCh1p12, ORF11 Nmag_4260
8481	8933	PhiH1_050	453	+	-	uncharacterized protein	PhiCh1p13, ORF12 Nmag_4261
8940	9542	PhiH1_055	603	+	*ada*	head-tail adaptor protein Ada	PhiCh1p14, ORF13 Nmag_4262
9539	9919	PhiH1_060	381	+	*hco*	head closure protein type 1 Hco	PhiCh1p15, ORF14 Nmag_4263
9921	10,202	PhiH1_065	282	+	-	uncharacterized protein	PhiCh1p16, ORF15 Nmag_4264
10,202	10,636	PhiH1_070	435	+	*nep*	probable neck protein type 1 Nep	PhiCh1p17, ORF16 Nmag_4265
10,643	11,239	PhiH1_075	597	+	*tco*	tail completion protein type 1 Tco	PhiCh1p18, ORF17 Nmag_4266
11,259	12,557	PhiH1_080	1299	+	*hp67*	tail sheath protein HP67	PhiCh1p19, ORF18 Nmag_4267
12,607	13,002	PhiH1_085	396	+	-	probable structural protein	PhiCh1p20, ORF19 Nmag_4268
13,006	13,407	PhiH1_090	402	+	-	uncharacterized protein	PhiCh1p21, ORF20 Nmag_4269
13,572	13,745	PhiH1_095	174	−	-	DUF4177 domain protein	[SEH60446.1]
13,792	16,581	PhiH1_100	2790	+	*tpm*	tape-measure tail protein Tpm	^4^ PhiCh1p23, ORF22 ^4^ PhiCh1p24, ORF23 Nmag_4272
16,583	17,104	PhiH1_105	522	+	-	uncharacterized protein	PhiCh1p25, ORF24 Nmag_4273
17,108	17,446	PhiH1_110	339	+	-	uncharacterized protein	PhiCh1p26, ORF25 Nmag_4274
17,450	18,298	PhiH1_115	849	+	-	uncharacterized protein	PhiCh1p27, ORF26 Nmag_4275
18,306	18,446	PhiH1_120	141	+	-	CxxC motif protein	[SEH61109.1]
18,443	18,988	PhiH1_125	546	+	-	uncharacterized protein	PhiCh1p29, ORF28 Nmag_4276
18,988	19,146	PhiH1_130	159	+	-	uncharacterized protein	-
19,143	19,508	PhiH1_135	366	+	-	virus-related protein	[AGM10900.1]
19,505	19,867	PhiH1_140	363	+	-	uncharacterized protein	PhiCh1p30, ORF29 Nmag_4277
19,874	21,148	PhiH1_145	1275	+	*bpj*	baseplate J family protein Bpj	PhiCh1p31, ORF30 Nmag_4278
21,135	22,277	PhiH1_150	1143	+	-	uncharacterized protein	PhiCh1p32, ORF31 Nmag_4279
22,295	22,678	PhiH1_155	384	+	-	virus-related protein	[AFH21897.1]
22,683	23,249	PhiH1_160	567	+	-	virus-related protein	[AFH21653.1]
23,252	25,504	PhiH1_165	2253	+	-	repeat-containing tail fibre protein	PhiCh1p37, ORF36 Nmag_4282 PhiCh1p35, ORF34 Nmag_4286
25,506	25,787	PhiH1_170	282	+	-	uncharacterized protein	Nmag_4285
25,825	26,499	PhiH1_175	675	+	*int1*	tyrosine integrase/recombinase Int1	PhiCh1p36, ORF35 Nmag_4284
26,490	26,792	PhiH1_180	303	−	-	uncharacterized protein	Nmag_4283
26,798	27,766	PhiH1_185	969	−	-	repeat-containing tail fibre protein ^2^	PhiCh1p37, ORF36 Nmag_4282 PhiCh1p35, ORF34 Nmag_4286
27,803	28,150	PhiH1_190	348	+	-	YncB-like endonuclease	[AGM11801.1]
28,153	28,386	PhiH1_195	234	+	-	virus-related protein	[AGC34510.1]
28,379	28,675	PhiH1_200	297	+	-	uncharacterized protein	[EMA49173.1]
28,682	28,783	PhiH1_205	102	+	-	uncharacterized protein	-
28,788	29,357	PhiH1_210	570	+	-	transmembrane domain protein	-
29,394	29,642	PhiH1_215	249	−	-	uncharacterized protein	-
29,651	29,941	PhiH1_220	291	−	-	uncharacterized protein	PhiCh1p40, ORF39 Nmag_4289
30,104	30,244	PhiH1_225	144	+	-	uncharacterized protein	-
30,250	30,414	PhiH1_230	165	+	-	uncharacterized protein	PhiCh1p44, ORF43 Nmag_4292
30,411	30,806	PhiH1_235	396	+	-	VapC family toxin	PhiCh1p45, ORF44 Nmag_4293
30,803	31,465	PhiH1_240	663	−	*int2*	tyrosine integrase/recombinase Int2	PhiCh1p46, ORF45 Nmag_4294
31,680	31,934	PhiH1_245	255	+	-	uncharacterized protein	-
31,939	32,271	PhiH1_250	333	+	-	uncharacterized protein	Nmag_4297
32,420	32,857	PhiH1_255	438	−	-	HNH-type endonuclease	PhiCh1p48, ORF47 Nmag_4296
32,854	33,255	PhiH1_260	402	−	-	uncharacterized protein	[ELY96531.1]
33,248	34,024	PhiH1_265	777	−	-	parA domain protein	PhiCh1p47, ORF46 Nmag_4295
34,161	34,430	PhiH1_270	270	−	*repR*	repressor protein RepR	^5^ PhiCh1p49, ORF48 ^5^ Nmag_4298 [ELZ06324.1]
34,730	35,071	PhiH1_275	342	+	-	uncharacterized protein	-
35,068	35,424	PhiH1_280	357	+	-	uncharacterized protein	PhiCh1p50, ORF49
35,381	38,167	PhiH1_285	2787	+	*repH*	plasmid replication protein RepH	^4^ PhiCh1p54, ORF53 ^4^ PhiCh1p55, ORF54 Nmag_4299
38,262	38,489	PhiH1_290	228	−	*imm*	probable immunity protein Imm	PhiCh1p56, ORF55 Nmag_4300
38,733	39,263	PhiH1_295	531	+	-	transcriptional regulator, PadR-like family	PhiCh1p57, ORF56 Nmag_4301
39,260	39,385	PhiH1_300	126	+	-	CxxC motif protein	-
39,382	39,978	PhiH1_305	597	+	-	uncharacterized protein	PhiCh1p59, ORF58 Nmag_4303
39,975	40,133	PhiH1_310	159	+	-	uncharacterized protein	-
40,153	40,902	PhiH1_315	750	+	*pcnA*	DNA polymerase sliding clamp PcnA	PhiCh1p60, ORF59 Nmag_4211
40,908	41,339	PhiH1_320	432	+	-	uncharacterized protein	PhiCh1p61, ORF60 Nmag_4212
41,339	41,554	PhiH1_325	216	+	-	uncharacterized protein	PhiCh1p62, ORF61 Nmag_4213
41,547	42,041	PhiH1_330	495	+	-	uncharacterized protein	-
42,098	42,490	PhiH1_335	393	+	*tnpA*	IS200-type transposase TnpA	[CAP12925.1]
42,492	43,748	PhiH1_340	1257	+	*tnpB*	IS1341-type transposase TnpB	[CAP12926.1]
43,808	44,014	PhiH1_345	207	+	-	uncharacterized protein	-
44,007	44,234	PhiH1_350	228	+	-	uncharacterized protein	PhiCh1p66, ORF65 Nmag_4217
44,231	44,656	PhiH1_355	426	+	-	CxxC motif protein	PhiCh1p68, ORF67 Nmag_4219
44,646	45,026	PhiH1_360	381	+	-	uncharacterized protein	PhiCh1p69, ORF68 Nmag_4220
45,023	45,646	PhiH1_365	624	+	-	HNH-type endonuclease	[KYG11427.1]
45,639	45,926	PhiH1_370	288	+	-	uncharacterized protein	PhiCh1p71, ORF70 Nmag_4222
45,919	46,350	PhiH1_375	432	+	-	DUF4326 domain protein	PhiCh1p72, ORF71 Nmag_4223
46,343	46,441	PhiH1_380	99	+	-	uncharacterized protein	-
46,438	46,884	PhiH1_385	447	+	-	CxxC motif protein	PhiCh1p74, ORF73 Nmag_4225
46,865	47,038	PhiH1_390	174	+	-	uncharacterized protein	^5^ PhiCh1p73, ORF72 ^5^ Nmag_4224
47,031	47,447	PhiH1_395	417	+	-	uncharacterized protein	-
47,440	47,739	PhiH1_400	300	+	-	NTPase protein	[PLX87675.1]
47,732	49,618	PhiH1_405	1887	+	*dcm5*	C-5 cytosine-specific DNA methylase Dcm5	^5^ PhiCh1p81, ORF80 [PCR88664.1]
49,611	49,931	PhiH1_410	321	+	-	uncharacterized protein	PhiCh1p82, ORF81 Nmag_4234
49,918	50,037	PhiH1_415	120	+	-	CxxC motif protein	-
50,091	51,452	PhiH1_420	1362	+	*yhdJ*	DNA methylase N-4/N-6 domain protein YhdJ	PhiCh1p83, ORF82 Nmag_4235
51,449	52,024	PhiH1_425	576	+	-	uncharacterized protein	PhiCh1p84, ORF83 Nmag_4236
52,021	52,791	PhiH1_430	771	+	-	uncharacterized protein	PhiCh1p85, ORF84 Nmag_4237
52,784	53,152	PhiH1_435	369	+	-	uncharacterized protein	PhiCh1p88, ORF87 Nmag_4240
53,145	53,504	PhiH1_440	360	+	-	uncharacterized protein	PhiCh1p89, ORF88 Nmag_4241
53,788	54,369	PhiH1_445	582	+	-	CxxC motif protein	PhiCh1p90, ORF89 Nmag_4242
54,403	54,771	PhiH1_450	369	+	-	uncharacterized protein	PhiCh1p91, ORF90 Nmag_4243
54,794	55,147	PhiH1_455	354	+	-	uncharacterized protein	-
55,144	55,401	PhiH1_460	258	+	-	transmembrane domain protein	PhiCh1p93, ORF92 Nmag_4244
55,394	55,729	PhiH1_465	336	+	-	transmembrane domain protein ^3^	PhiCh1p94, ORF93 Nmag_4245
55,794	57,053	PhiH1_470	1260	+	*ycdA*	DNA methylase N-4/N-6 domain protein YcdA	PhiCh1p95, ORF94 Nmag_4246
57,046	57,564	PhiH1_475	519	+	-	uncharacterized protein	PhiCh1p96, ORF95 Nmag_4247
57,621	57,830	PhiH1_480	210	+	-	CxxC motif protein	PhiCh1p98, ORF97 Nmag_4249
57,827>	<63	PhiH1_485	309	+	*terS*	terminase small subunit TerS	PhiCh1p01, ORF98 Nmag_4250

^1^ PhiCh1/pNMAG03 homologs of phiH1 proteins show BLASTp E-values < 10^−20^. For phiCh1 proteins, both the PhiCh1p and originally assigned ORF codes (ORF for open reading frame) are shown (e.g., PhiCh1p02, ORF1). Codes starting with ORF represent the original annotation of the phiCh1 genome [17] (GB accession AF440695.1); and codes starting with PhiCh1p represent the RefSeq version of the annotation of the same genome sequence (GB accession NC_004084). The number shift is due to the *terS* gene, the N-terminal part being encoded at the end of the genome, and the C-terminal part at its beginning. This ORF is complete in the provirus state due to circularization and in the linear virus state due to terminal redundancy. This gene is *ORF98* in the original annotation and PhiCh1p01 in the RefSeq annotation. Codes starting with Nmag_ represent the annotation of the *Natrialba magadii* plasmid pNMAG03 [20] (accession CP001935.1). The point of ring opening in pNMAG03 was set between Nmag_4303 and Nmag_4211. Codes in square brackets represent NCBI accessions referring to homologous proteins (BLASTp E-values ≤ 10^−11^), which are from other sources. ^2^ Gene PhiH1_185 is encoded on an invertible segment. In the current sequence version, it is inactivated because it is uncoupled from a start codon. By genome inversion, it becomes activated while its partner gene PhiH1_165 becomes inactivated. Overall, this results in tail fibre protein switching. ^3^ This protein (PhiH1_465) has three predicted transmembrane domains and has been suspected to function as a holin [18]. ^4^ In these cases, the phiCh1 gene is split into two CDS but is continuous in phiH1. ^5^ These proteins are more distantly related (show less than 39% sequence identity or fall above BLASTp E-values of 10^−20^). In these cases, a similar genetic context supports their stated relationship.

**Table 2:** CRISPR spacers matching phiH1.

No.	CRISPR Spacer Matches to phiH1 ^1^	Translation ^2^
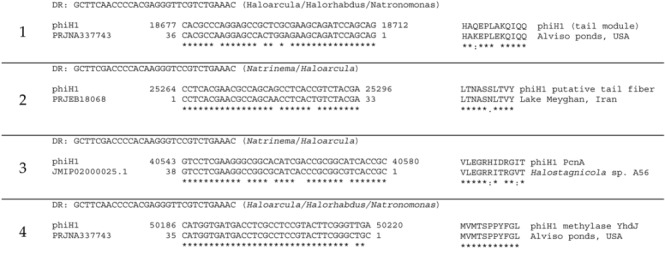		

^1^ The matching spacer sequences were found in the following NCBI bioprojects using the crass program: PRJNA337743, (SRA SRR4030040; Alviso Ponds, San Francisco, CA, USA; metagenome); PRJNA245787 (*Halostagnicola* sp. A56 26 genome; Andaman Islands, India); PRJEB18068 (Lake Meyghan, Iran; metagenome). Aligned sequences show nt positions for phiH1, and asterisks indicated identical bases. DR: direct repeat (with haloarchaea containing most closely matching DR shown in brackets). ^2^ Symbols under alignment (*:.) indicate identical, similar and weakly similar residues, respectively (based on Gonnet PAM 250 matrix).

## Supplementary Materials

The following are available online at http://www.mdpi.com/2073-4425/9/10/493/s1, Figure S1: Original phiH1 restriction map compared to in silico map from genome sequence. Figure S2: Dot plot sequence comparison of phiH1 and phiCh1 genomes.
