# Supplementary material for: Complete Genome Sequence of the Model Halovirus PhiH1 (ΦH1)
Source: Genes (Basel). 2018 Oct 12;9(10):493. doi: 10.3390/genes9100493 (PMC6210493; doi:10.3390/genes9100493)
Supplement: Supplementary file 1 [file genes-09-00493-s001.pdf]

genes

MDPI

Supplementary Materials

Figure S1.

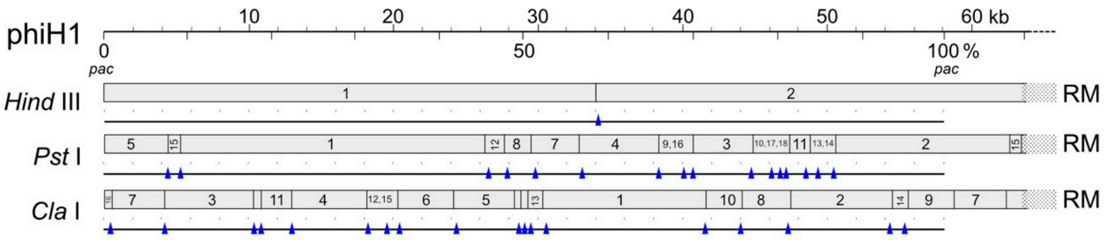

Figure S1. Original phiH1 restriction map compared to *in silico* map determined from the genome sequence. The published phiH1 restriction map (RM), modified from Figure 4 of [1], is compared to an *in silico* map based on the complete genome sequence. The top scale and grey-shaded bars are traced reproductions of the original viral DNA restriction maps, including the fragment numbers [1]. Below each of these are the corresponding *in silico* restriction maps (black lines, blue triangles), determined from the complete genome sequence. Underneath each of these are tick marks every 2 kb. Enzyme names are shown at the left, and *pac* sites indicated at the 0 and 100% marks. Maps extend beyond 100% to indicate the terminal redundancy and circularly-permuted nature of the virion DNA. A distinct subpopulation of virus particles contain genomes starting at the *pac* site, which leads to faint but readily detectable restriction fragments assigned to equivalent genome positions (e.g. fragments 5 and 2 for PstI, or fragments 16 and 9 for ClaI).

Reference

1. Schnabel, H.; Schramm, E.; Schnabel, R.; Zillig, W. Structural variability in the genome of phage ΦH of *Halobacterium halobium*. Mol. Gen. Genet. 1982, 188, 370-377.

Figure S2.

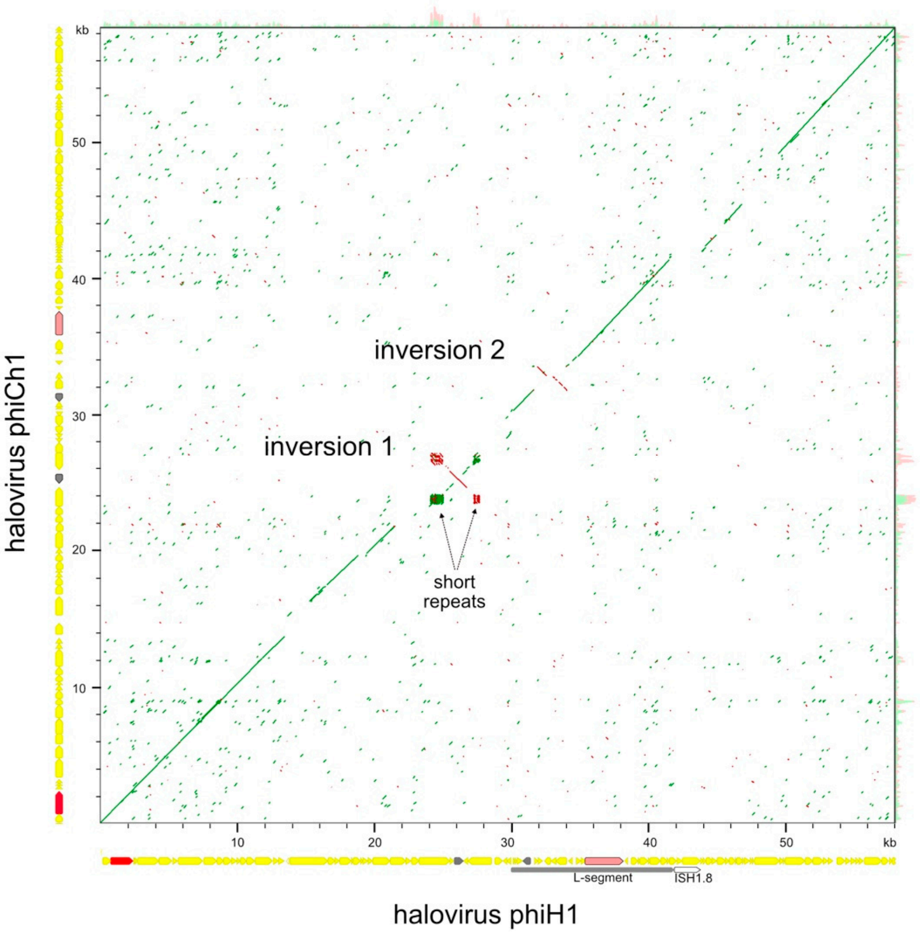

Figure S2. Dot plot sequence comparison of phiH1 and phiCh1 genomes. Genomes were aligned using the YASS aligner (http://bioinfo.lifl.fr/yass/). Alignment in forward direction is green, and in reverse direction is red. Along the axes are arrows representing coding sequences, with terminase genes (*terL*) coloured red, integrase/recombinase genes in grey, and repH genes in pink. The short repeats within inversion 1 result in high-enough DNA similarity to be detected in both orientations.

© 2018 by the authors. Submitted for possible open access publication under the terms and conditions of the Creative Commons Attribution (CC BY) license (http://creativecommons.org/licenses/by/4.0/).
